# Epidemiology of Enteroaggregative, Enteropathogenic, and Shiga Toxin–Producing *Escherichia coli* Among Children Aged <5 Years in 3 Countries in Africa, 2015–2018: Vaccine Impact on Diarrhea in Africa (VIDA) Study

**DOI:** 10.1093/cid/ciad035

**Published:** 2023-04-19

**Authors:** John B Ochieng, Helen Powell, Ciara E Sugerman, Richard Omore, Billy Ogwel, Jane Juma, Alex O Awuor, Samba O Sow, Doh Sanogo, Uma Onwuchekwa, Adama Mamby Keita, Awa Traoré, Henry Badji, M Jahangir Hossain, Joquina Chiquita M Jones, Irene N Kasumba, Dilruba Nasrin, Anna Roose, Yuanyuan Liang, Leslie P Jamka, Martin Antonio, James A Platts-Mills, Jie Liu, Eric R Houpt, Eric D Mintz, Elizabeth Hunsperger, Clayton O Onyango, Nancy Strockbine, Marc-Alain Widdowson, Jennifer R Verani, Sharon M Tennant, Karen L Kotloff

**Affiliations:** Kenya Medical Research Institute, Center for Global Health Research, Kisumu, Kenya; Center for Vaccine Development and Global Health, University of Maryland School of Medicine, Baltimore, Maryland, USA; Department of Pediatrics, University of Maryland School of Medicine, Baltimore, Maryland, USA; Division of Foodborne, Waterborne, and Environmental Diseases, Centers for Disease Control and Prevention, Atlanta, Georgia, USA; Kenya Medical Research Institute, Center for Global Health Research, Kisumu, Kenya; Kenya Medical Research Institute, Center for Global Health Research, Kisumu, Kenya; Kenya Medical Research Institute, Center for Global Health Research, Kisumu, Kenya; Kenya Medical Research Institute, Center for Global Health Research, Kisumu, Kenya; Centre pour le Développement des Vaccins du Mali, Bamako, Mali; Centre pour le Développement des Vaccins du Mali, Bamako, Mali; Centre pour le Développement des Vaccins du Mali, Bamako, Mali; Centre pour le Développement des Vaccins du Mali, Bamako, Mali; Centre pour le Développement des Vaccins du Mali, Bamako, Mali; Medical Research Council Unit, The Gambia at the London School of Hygiene & Tropical Medicine, Banjul, The Gambia; Medical Research Council Unit, The Gambia at the London School of Hygiene & Tropical Medicine, Banjul, The Gambia; Medical Research Council Unit, The Gambia at the London School of Hygiene & Tropical Medicine, Banjul, The Gambia; Center for Vaccine Development and Global Health, University of Maryland School of Medicine, Baltimore, Maryland, USA; Department of Medicine, University of Maryland School of Medicine, Baltimore, Maryland, USA; Center for Vaccine Development and Global Health, University of Maryland School of Medicine, Baltimore, Maryland, USA; Department of Medicine, University of Maryland School of Medicine, Baltimore, Maryland, USA; Center for Vaccine Development and Global Health, University of Maryland School of Medicine, Baltimore, Maryland, USA; Department of Pediatrics, University of Maryland School of Medicine, Baltimore, Maryland, USA; Center for Vaccine Development and Global Health, University of Maryland School of Medicine, Baltimore, Maryland, USA; Department of Epidemiology and Public Health, University of Maryland School of Medicine, Baltimore, Maryland, USA; Center for Vaccine Development and Global Health, University of Maryland School of Medicine, Baltimore, Maryland, USA; Department of Medicine, University of Maryland School of Medicine, Baltimore, Maryland, USA; Medical Research Council Unit, The Gambia at the London School of Hygiene & Tropical Medicine, Banjul, The Gambia; Division of Infectious Diseases and International Health, Department of Medicine, University of Virginia, Charlottesville, Virginia, USA; Division of Infectious Diseases and International Health, Department of Medicine, University of Virginia, Charlottesville, Virginia, USA; Department of Microbial Surveillance and Biosafety, School of Public Health, Qingdao University, Qingdao, China; Division of Infectious Diseases and International Health, Department of Medicine, University of Virginia, Charlottesville, Virginia, USA; Division of Foodborne, Waterborne, and Environmental Diseases, Centers for Disease Control and Prevention, Atlanta, Georgia, USA; Division of Global Health Protection, Centers for Disease Control and Prevention, Nairobi, Kenya; Division of Global Health Protection, Centers for Disease Control and Prevention, Nairobi, Kenya; Division of Foodborne, Waterborne, and Environmental Diseases, Centers for Disease Control and Prevention, Atlanta, Georgia, USA; Division of Global Health Protection, Centers for Disease Control and Prevention, Nairobi, Kenya; Division of Global Health Protection, Centers for Disease Control and Prevention, Nairobi, Kenya; Center for Vaccine Development and Global Health, University of Maryland School of Medicine, Baltimore, Maryland, USA; Department of Medicine, University of Maryland School of Medicine, Baltimore, Maryland, USA; Center for Vaccine Development and Global Health, University of Maryland School of Medicine, Baltimore, Maryland, USA; Department of Pediatrics, University of Maryland School of Medicine, Baltimore, Maryland, USA; Department of Medicine, University of Maryland School of Medicine, Baltimore, Maryland, USA; Department of Epidemiology and Public Health, University of Maryland School of Medicine, Baltimore, Maryland, USA

**Keywords:** diarrheagenic *E*. *coli*, EPEC, EAEC, STEC, pediatric diarrhea

## Abstract

**Background:**

To address knowledge gaps regarding diarrheagenic *Escherichia coli* (DEC) in Africa, we assessed the clinical and epidemiological features of enteroaggregative *E*. *coli* (EAEC), enteropathogenic *E. coli* (EPEC), and Shiga toxin–producing *E. coli* (STEC) positive children with moderate-to-severe diarrhea (MSD) in Mali, The Gambia, and Kenya.

**Methods:**

Between May 2015 and July 2018, children aged 0–59 months with medically attended MSD and matched controls without diarrhea were enrolled. Stools were tested conventionally using culture and multiplex polymerase chain reaction (PCR), and by quantitative PCR (qPCR). We assessed DEC detection by site, age, clinical characteristics, and enteric coinfection.

**Results:**

Among 4840 children with MSD and 6213 matched controls enrolled, 4836 cases and 1 control per case were tested using qPCR. Of the DEC detected with TAC, 61.1% were EAEC, 25.3% atypical EPEC (aEPEC), 22.4% typical EPEC (tEPEC), and 7.2% STEC. Detection was higher in controls than in MSD cases for EAEC (63.9% vs 58.3%, *P* < .01), aEPEC (27.3% vs 23.3%, *P* < .01), and STEC (9.3% vs 5.1%, *P* < .01). EAEC and tEPEC were more frequent in children aged <23 months, aEPEC was similar across age strata, and STEC increased with age. No association between nutritional status at follow-up and DEC pathotypes was found. DEC coinfection with *Shigella*/enteroinvasive *E. coli* was more common among cases (*P* < .01).

**Conclusions:**

No significant association was detected between EAEC, tEPEC, aEPEC, or STEC and MSD using either conventional assay or TAC. Genomic analysis may provide a better definition of the virulence factors associated with diarrheal disease.

While residing in the human intestine as commensal flora, strains of *Escherichia coli* have acquired the ability to cause diarrheal disease via the horizontal transfer of virulence genes from cohabitating intestinal bacteria [1]. Over time, transformed strains with a survival advantage have propagated within different *E. coli* phylogenetic lineages to become pathotypes that produce a broad spectrum of diarrheal diseases, sometimes with severe consequences [2]. Known as diarrheagenic *E. coli* (DEC), 5 distinct categories have been identified: enteroaggregative *E. coli* (EAEC), enteropathogenic *E. coli* (EPEC), enterotoxigenic *E. coli* (ETEC), Shiga toxin–producing *E. coli* (STEC)/enterohemorrhagic *E. coli*, and enteroinvasive *E. coli* (EIEC). The pathogenicity of a sixth category, diffusely adherent *E. coli*, remains uncertain [1]. EPEC is further classified as typical (tEPEC) and atypical (aEPEC) based on the detection of bundle-forming pilus (*bfpA*) and/or *E. coli* attaching and effacing (*eae*) virulence genes, as described below [1]. In general, strains within each pathotype share virulence factors that produce similar clinical manifestations and pathologic features, often with characteristic host predilections, transmission dynamics, epidemiology, and disease burden. Together, they are among the leading causes of diarrhea-associated morbidity and mortality in children aged <5 years in low- and middle-income countries (LMICs) [3, 4].

Traditionally, laboratory diagnosis of DEC required isolation of *E. coli* colonies from stool culture followed by detection of distinguishing pathotype-associated features using either phenotypic assays such as microscopy, serology, and antigen detection or molecular tests such as gene probe and polymerase chain reaction (PCR) [5]. The advent of molecular panels such as the quantitative PCR (qPCR)–based TaqMan Array card (TAC) introduced a tool with many advantages for performing diarrheal disease research, including high sensitivity, rapid throughput, and the ability to contemporaneously test stool samples directly for a broad array of pathogens. In addition, qPCR allows determination of pathogen-specific cycle threshold (Ct) values that can distinguish cases from controls under the assumption that symptomatic infections have higher pathogen burdens [6].

The Global Enteric Multicenter Study (GEMS), a case-control study of medically attended moderate-to-severe diarrhea (MSD) among children aged <5 years living in LMICs in Asia and Africa, identified DEC using conventional multiplex PCR to test *E. coli* isolated from stool samples [3]. ETEC that produced heat-stable toxin with or without heat-labile toxin was found to be a major cause of MSD. STEC was the least frequent DEC in contrast to its predominance in high-resource settings where it causes diarrhea associated with hemorrhagic colitis and hemolytic uremic syndrome [7]. Whereas tEPEC was significantly associated with MSD in children aged <2 years in Kenya and death in infants aged <1 year, aEPEC was not associated with MSD or death at any site [3, 8]. GEMS reported a high prevalence of EAEC among symptomatic and asymptomatic children, with inconsistent associations with diarrhea [3, 9, 10].

A retrospective etiological reanalysis of a subset of samples from cases and controls who participated in GEMS using TAC qPCR demonstrated an increase in the total pathogen-specific attributable diarrheal burden from 51.5% using culture plus multiplex PCR to 89.3% using TAC, suggesting that conventional methods underestimate the prevalence of many pathogens [11]. To update our understanding of the epidemiology of DEC among children with and without diarrhea, with a focus on settings where rotavirus vaccine introduction may have altered the landscape of enteric pathogens, we examined data from the Vaccine Impact on Diarrhea in Africa (VIDA) study, a follow-on study to GEMS at 3 sites in sub-Saharan Africa [12]. In VIDA, stool samples from children with MSD and their controls were contemporaneously tested using both conventional multiplex PCR and TAC, allowing for a comparison of the associations between DEC pathotypes and MSD using both diagnostic approaches. In addition, we determined whether DEC coinfection with other enteric pathogens increased the severity of disease, as has been reported elsewhere [13]. We specifically focused on EPEC, EAEC, and STEC, whose role in diarrheal disease has not been clearly elucidated in Africa. EIEC cannot be distinguished from *Shigella* using TAC and so was not included in this analysis. Because of its considerable burden, ETEC will be the subject of a separate article.

## METHODS

### Study Design and Participants

Three sites (Bamako, Mali; Basse and Bansang, The Gambia; and Siaya County, Kenya) were selected from African countries with high childhood mortality [3] that had introduced rotavirus vaccine. VIDA participants resided within a demographic surveillance system (DSS) catchment area.

For 36 months at each site between May 2015 and July 2018, children in 3 age strata (0–11 months, 12–23 months, and 24–59 months) who sought care at health facilities serving the DSS were assessed for MSD as previously described [3]. MSD was defined as ≥3 loose stools within the last 24 hours [14] plus ≥1 of the following: sunken eyes, skin tenting, dysentery, required intravenous rehydration, or hospitalization within 7 days of diarrhea onset. Within 2 weeks of enrolling each MSD case, 1–3 diarrhea-free controls matched by age, sex, and neighborhood were randomly selected from the site's DSS database and enrolled at home, as described [3]. A follow-up home visit was made to every enrolled child 50–90 days after enrollment (average 60).

Demographic, epidemiologic, and clinical data were collected, and anthropometry was performed for all cases and controls at the enrollment and follow-up visits as described [15]. To determine the duration of diarrhea, caregivers recorded the occurrence of diarrhea daily for 14 days after enrollment using a simple pictorial memory aid [16] that was reviewed with the caretaker and collected at the follow-up visit. The total duration of diarrhea was defined as the days with diarrhea prior to enrollment plus the 14 days post-enrollment recorded on the memory aid (Supplementary Figure 1).

### Sample Collection and Laboratory Analysis

Each case and control provided at least 3 g of fresh whole stool that was placed in cold storage within 1 hour of production. The whole stool was swabbed or a rectal swab was obtained if antibiotics were to be administered [17]. Swabs for culture of *E. coli* were placed in Cary–Blair transport media (Oxoid/REMEL, Inc, Lenexa, KS) within 6 hours of production and transported to the microbiology laboratory.

### Diarrheagenic *E. coli* Detection Using Conventional Culture Plus PCR Methods

Immediately upon arrival at the laboratory, the swab was inoculated on culture media and incubated aerobically at 35°C–36°C for 18–24 hours. Three colonies of *E. coli* from every stool were pooled for PCR analysis. Primers targeting specific genes for detection of EPEC, EAEC, and STEC were used as previously described [17, 18]. In brief, typical and atypical EPEC were identified by primers targeting *bfp*A and *eae* genes and classified as tEPEC (*bfp*A detected with or without *eae*) and as aEPEC (*eae* detected without *bfp*A, *stx*_1_, or *stx*_2_). EAEC presence was defined by detection of *aaiC* and/or *aatA*. STEC was defined by amplification of *stx*_1_ and/or *stx*_2_ (regardless of *eae*) without *bfpA*.

### Diarrheagenic *E. coli* Pathotype Analysis Using TAC qPCR

Stool specimens were aliquoted and stored at −80°C until extraction. Total nucleic acid (TNA) was extracted from 200 mg of the whole stool specimen using the QIAamp Fast DNA stool mini kit (Qiagen, Hilden, Germany) with a modification that involved addition of glass beads to weighed stool before the addition of lysis buffer, then bead-beating to obtain a homogeneous mixture for TNA extraction [19]. The TNAs were tested on a real-time qPCR platform using TAC (Thermo Fisher, Carlsbad, CA), which amplifies nucleic acid for 30 enteropathogens plus multiple genotypes of several pathogens. The amplification curves were analyzed on Vii7 software (version 1.2.4) [11]. The primer (*vide supra*) is further described by Liu et al [19]. Samples with a primer target quantification Ct <35 were considered positive for that pathogen.

### Data Analyses

First, we compared the proportion of cases with detection by conventional and TAC methods for each pathogen in case or control specimens. The remainder of the analyses only used the TAC results. We compared the Ct values in cases and controls using a Wilcoxon rank sum test to assess the relative pathogen quantities. We calculated the positivity proportions among cases and controls by demographic characteristics, site, clinical characteristics, severity, and rates of coinfection with other enteric pathogens detected using TAC, including adenovirus 40/41, *Aeromonas* spp., astrovirus, toxigenic *Bacillus fragilis*, *Campylobacter* spp., *Cryptosporidium* spp., *Enterocytozoon bieneusi*, *Helicobacter pylori*, norovirus GI, norovirus GII, *Plesiomonas* spp., rotavirus, *Salmonella* spp., sapovirus, *Shigella* spp./EIEC, and heat stable– or heat labile–producing enterotoxigenic *E. coli*. *χ*^2^ tests were used to compare categorical variables; a *P* value <.05 was considered statistically significant.

We analyzed MSD cases to determine whether EPEC, EAEC, or STEC was associated with stunting, as measured at the enrollment and follow-up visit as described in Supplementary Table 1 and [15]. Stunting was defined as a height/length-for-age *z* score >2 standard deviations below the World Health Organization child growth standard median [20]. We initially compared stunting at follow-up between positive vs negative MSD cases using a *χ*^2^ test. Thereafter, we used propensity score matching to limit potential selection bias from the design of the original case/control study (Supplementary Table 1). We report the average treatment effect for the treated, that is, the difference in expected growth if those who were infected actually had not been infected with associated 95% confidence intervals and *P* values estimated via bootstrapping.

Statistical significance was defined as a *P* value < .05, and all analyses were performed using Stata/SE version 16.

### Ethical Review

This project was approved by the institutional review boards of the University of Maryland, Baltimore (HP-00062472); the US Centers for Disease Control and Prevention (CDC), Atlanta, Georgia (reliance agreement, CDC protocol number 6729); The Gambia government/Medical Research Council/Gambia at the London School of Hygiene and Tropical Medicine (1409); the Comité d'Ethique de la Faculté de Médecine, de Pharmacie, et d'Odonto-Stomatologie, Bamako, Mali (no number); and the Kenya Medical Research Institute Scientific and Ethics Review Unit in Siaya County, Kenya (SSE 2996). Written informed consent was obtained from the parent or primary caretaker of each child who met eligibility criteria before any research activities were performed.

## RESULTS

Collectively, 4840 MSD cases and 6213 matched controls were enrolled across the 3 sites. The characteristics of cases and controls enrolled in the VIDA study are described elsewhere [12].

**Figure 1.**
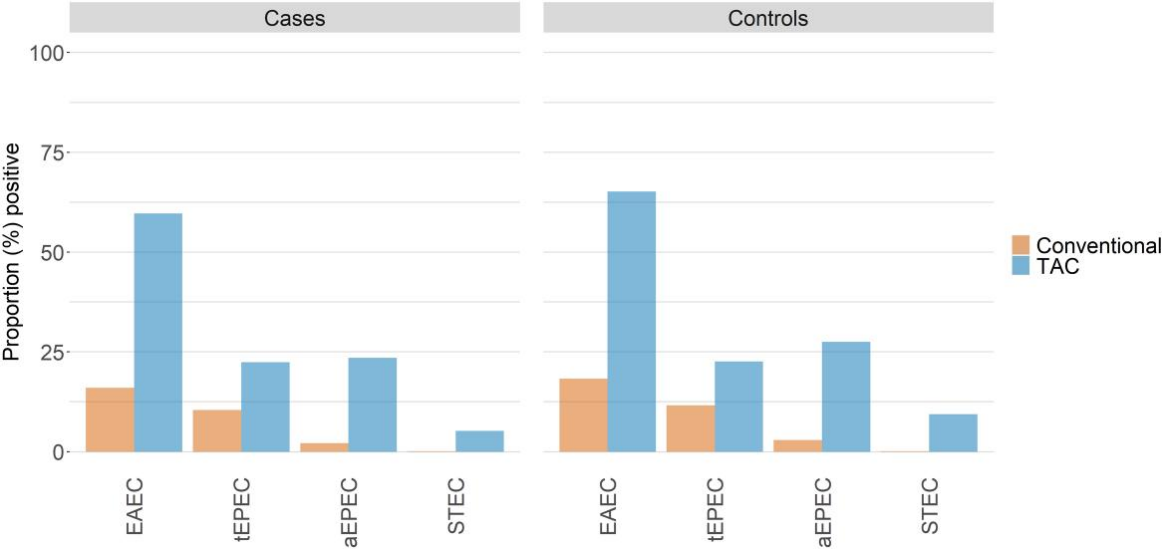
Comparison of TAC vs conventional laboratory methods for detection of diarrheagenic *Escherichia coli* (EAEC, tEPEC, aEPEC, and STEC) among case and control children aged <5 years. Abbreviations: aEPEC, atypical enteropathogenic *E. coli*; EAEC, enteroaggregative *E. coli*; STEC, Shiga toxin–producing *E. coli*; tEPEC, typical enteropathogenic *E. coli*; TAC, TaqMan Array card.

When case and control children evaluated using both TAC and conventional assays (n = 9672) were compared (Figure 1), the proportion who had pathogens detected using TAC was more than 2-fold higher than the proportion detected using conventional methods. Moreover, nearly all individual DEC pathotypes that were positive by conventional methods were also positive by TAC. Specifically, the proportion positive by conventional methods who had negative TAC results was only 4.4%, 1.3%, and 0.1% for EAEC, aEPEC, and STEC, respectively, among cases and 3.8%, 2.4%, and 0.1%, respectively, among controls (Supplementary Table 2). The exception was tEPEC, for which 5.7% of cases and 8.3% of controls were positive by conventional methods but negative by TAC. Nonetheless, the increased ability to detect DEC was harmonious across cases and controls, so that the relative isolation rates among cases and controls were similar regardless of method (Table 1). Likewise, the distribution of the Ct values for specific DEC pathotypes was similar in both cases and controls with the exception of tEPEC, which had significantly lower Ct values in cases than in controls (*P* = .0001; Supplementary Figure 2, Supplementary Table 3).

**Table 1.** Detection of Diarrheagenic *Escherichia coli* Pathotypes Using Quantitative Polymerase Chain Reaction (PCR) and Conventional Culture Plus Multiplex PCR From Moderate-to-Severe Diarrhea Cases and Their Matched Controls

	Quantitative Polymerase Chain Reaction		Conventional		
Pathotype	No. Positive	(%)	No. Positive	(%)	Fold-Rise in Detection^a^
Enteroaggregative *Escherichia coli*					
ȃCases (n = 4719)	2817	(59.7)	755	(16.0)	3.7
ȃControls (n = 4733)	3088	(65.2)	866	(18.3)	3.6
Typical enteropathogenic *E. coli*					
ȃCases (n = 4790)	1075	(22.4)	496	(10.4)	2.2
ȃControls (n = 4795)	1086	(22.6)	554	(11.6)	2.0
Atypical enteropathogenic *E. coli*					
ȃCases (n = 4788)	1127	(23.5)	101	(2.1)	11.2
ȃControls (n = 4789)	1318	(27.5)	139	(2.9)	9.5
Shiga toxin–producing *E. coli*					
ȃCases (n = 4792)	247	(5.2)	4	(0.1)	61.8
ȃControls (n = 4786)	451	(9.4)	5	(0.1)	90.2

Calculated as the number detected using quantitative polymerase chain reaction divided by the number detected using the conventional assay, according to cases or control status, by pathotype.

**Figure 2.**
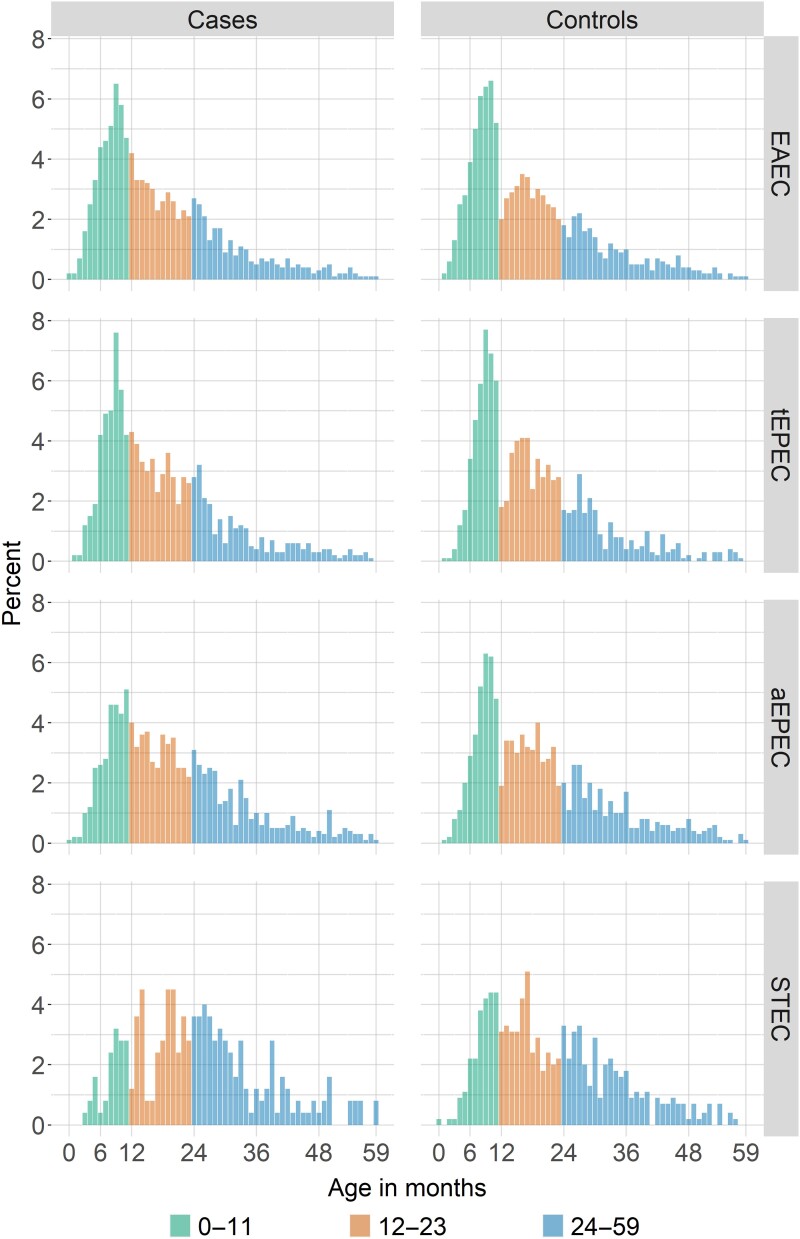
Proportions of EAEC, tEPEC, aEPEC, and STEC detected using TAC among case and control children aged <5 years. Abbreviations: aEPEC, atypical enteropathogenic *E. coli*; EAEC, enteroaggregative *E. coli*; STEC, Shiga toxin–producing *E. coli*; tEPEC, typical enteropathogenic *E. coli*; TAC, TaqMan Array card.

The distribution of EAEC, EPEC, and STEC pathotypes overall and by site, age, and sex according to case vs control status is presented in Table 2. EAEC was the most common pathotype detected (61.1%) followed by EPEC (aEPEC 25.3% and tEPEC 22.4%), with STEC being the least common (7.2%). The proportions of EAEC, aEPEC, and STEC were significantly lower in cases than in controls (58.3% vs 63.9%, 23.3% vs 27.3%, and 5.1% vs 9.3%, respectively; all *P* <.001). In contrast, tEPEC was similar in both cases and controls (22.3% vs 22.5%, *P* > .05). When *eae* was included in the definition of STEC, 3.3% of cases and 7.0% of controls met the TAC definition of positive, so the lack of association with MSD was unchanged (Table 2, Supplementary Table 4). The frequencies of *stx*_1_ and *stx*_2_ were similar within the case and control groups, and both genotypes were similar or less common in cases compared with controls. Among both cases and controls, the prevalence of EAEC and tEPEC declined with age, while the proportion of children with aEPEC was similar across ages and the prevalence of STEC increased with age. STEC frequency appeared to peak in MSD children aged 12–30 months (Figure 2).

**Table 2.** Proportion of Enteropathogenic *Escherichia coli*, Enteroaggregative *E. coli,* and Shiga Toxin–Producing *E. coli* Pathotypes Positive by TaqMan Array Card System Quantitative Polymerase Chain Reaction With Cycle Threshold <35, Overall and Among Case and Control Children Aged <5 Years

No. Positive for Each Pathotype, 9672 Tested (%)^a^	Enteroaggregative *Escherichia coli* n = 5908 (61.1%)^a^		Typical Enteropathogenic *E. coli* n = 2163 (22.4%)^a^		Atypical Enteropathogenic *E. coli* n = 2448 (25.3%)^a^		Shiga Toxin–Producing *E. coli* n = 698 (7.2%)^a^	
No. of cases and controls positive by pathotype (%)	Case n = 2818/4836 (58.3%)	Control n = 3090/4836 (63.9%)^b^	Case n = 1077/4836 (22.3%)	Control n = 1086/4836 (22.5%)	Case n = 1127/4836 (23.3%)^b^	Control n = 1321/4836 (27.3%)	Case n = 247/4836 (5.1%)^b,c^	Control n = 451/4836 (9.3%)^d^
Site								
ȃThe Gambia	983 (34.9%)	1085 (35.1%)	427 (39.7%)	421 (38.8%)	365 (32.4%)	462 (35.0%)	79 (32.0%)	154 (34.2%)
ȃMali	956 (33.9%)	1039 (33.6%)	404 (37.5%)	408 (37.6%)	345 (30.6%)	422 (32.0%)	55 (22.3%)	91 (20.2%)
ȃKenya	879 (31.2%)	966 (31.3%)	246 (22.8%)	257 (23.7%)	417 (37.0%)	437 (33.1%)	113 (45.8%)	206 (45.7%)
Age group, mo								
ȃ0–11	1122 (39.8%)	1259 (40.7%)	394 (36.6%)	413 (38.0%)	328 (29.1%)	438 (33.2%)	38 (15.4%)	108 (24.0%)
ȃ12–23	952 (33.8%)	1022 (33.1%)	396 (36.8%)	401 (36.9%)	420 (37.3%)	478 (36.2%)	84 (34.0%)	161 (35.7%)
ȃ24–59	744 (26.4%)	809 (26.2%)	287 (26.7%)	272 (25.1%)	379 (33.6%)	405 (30.7%)	125 (50.6%)	182 (40.4%)
Sex								
ȃMale	1534 (54.4%)	1666 (53.9%)	581 (54%)	581 (53.5%)	615 (54.6%)	699 (52.9%)	136 (55.1%)	241 (53.4%)
ȃFemale	1284 (45.6%)	1424 (46.1%)	496 (46.1%)	505 (46.5%)	512 (45.4%)	622 (47.1%)	111 (44.9%)	210 (46.6%)

Positive defined by TaqMan Array Card system quantitative polymerase chain reaction (cycle threshold <35).

The proportion of the *E. coli* pathotype was significantly higher in controls compared with cases for enteroaggregative *E. coli* (63.9 vs 58.3%, *P* = .0004), atypical enteropathogenic *E. coli* (27.3 vs 23.3%, *P* < .0001), and Shiga toxin–producing *E. coli* (STEC; 9.3 vs 5.1%, *P* < .0001).

Of the 247 moderate-to-severe diarrhea cases in whom STEC was identified, 160 tested positive for the *eae* gene (64.8%), or 3.3% of cases. The distribution of the Shiga toxin genotype among the 247 STEC-positive cases was as follows: 93 had *stx*1 alone (37.3%, or 1.9% of cases), 91 had *stx*2 alone (36.8%, or 1.9% of cases), and 63 had both (25.5%, or 1.3% of cases).

Of the 451 controls in whom STEC was identified, 338 tested positive for the *eae* gene (74.9%), or 7.0% of controls. The distribution of the Shiga toxin genotype among the 451 STEC-positive controls was as follows: 147 had *stx*1 alone (32.6%, or 3.0% of controls), 159 had *stx*2 alone (35.3%, or 3.3% of controls), and 145 had both (32.2%, or 3.0% of controls).

Caretaker report of the presence of ruminant animals (cows, goats, or sheep) in the child's compound was explored as a potential source of infection with STEC. STEC positivity in the child's stool was significantly associated with ruminant exposure (*P* < .01) for both cases and controls across all age groups. More than 73% of STEC-positive case and control children had a ruminant animal present in their domiciles compared with 60% for STEC-negative cases and controls (Table 3).

**Table 3.** Proportion of Shiga Toxin–Producing *Escherichia coli* Among Children Living With and Without Ruminant Animals in the Compound by Case/Control Status and by Age

	Cases			Controls		
	STEC-Positive n = 247	STEC-Negative n = 4552	*P* Value	STEC-Positive n = 451	STEC-Negative n = 4342	*P* Value^a^
No. (%) positive or negative for STEC, case/control status, with ruminants living in the compound (cow, goat, or sheep)						
Ruminants present	187 (75.7%)	2728 (59.9%)	**<**.**001**	332 (73.6%)	2644 (60.9%)	**<**.**001**
Age group, mo						
ȃ0–11	31/38 (81.6%)	1040/1670 (62.3%)	.**024**	80/108 (74.1%)	962/1592 (60.4%)	.**005**
ȃ12–23	64/84 (76.2%)	934/1592 (58.7%)	.**002**	129/161 (80.1%)	943/1520 (62.0%)	**<**.**001**
ȃ24–59	92/125 (73.6%)	754/1290 (58.4%)	.**001**	123/182 (67.6%)	739/1230 (60.1%)	0.067

Positive defined as Ct <35; negative defined as Ct ≥35.Abbreviation: STEC, Shiga toxin–producing *E. coli*; Ct, cycle threshold.

*χ* ^2^ tests were used to estimate *P* values; significant values are shown in bold.

Of the 4840 MSD cases enrolled in VIDA, 223 had missing length/height measurements and 14 had implausible values, reducing the analytical dataset to 4603 children with MSD for the stunting analysis. Although crude unadjusted analyses showed several significant associations (Supplementary Table 5), no DEC pathotype was associated with linear growth faltering at the follow-up visit 2–3 months after enrollment when propensity score matching was used (Supplementary Tables 6 and 7).

**Figure 3.**
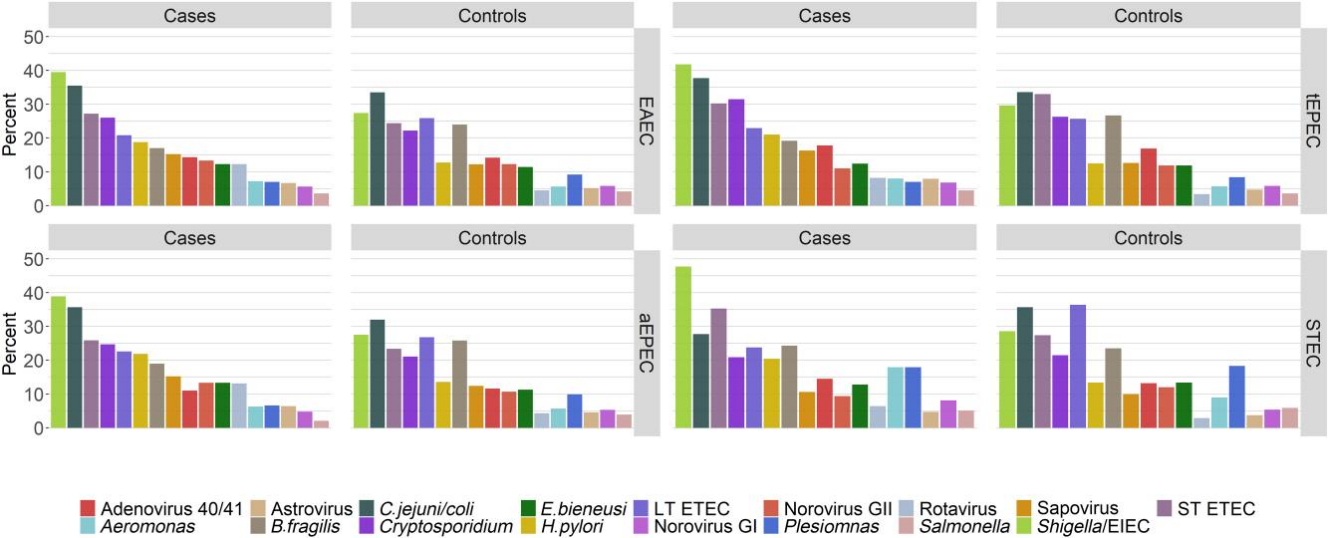
Frequency of coinfection in cases and controls with most common enteric pathogens in children aged <5 years. Abbreviations: aEPEC, atypical enteropathogenic *Escherichia coli*; EAEC, enteroaggregative *E. coli*; LT ETEC, Heat-labile toxin-producing enterotoxigenic *E. coli*; ST ETEC, Heat-stable toxin-producing enterotoxigenic *E. coli*, STEC, Shiga toxin–producing *E. coli*; tEPEC, typical enteropathogenic *E. coli*.

DEC pathotypes were found more often in MSD episodes in which there was a mixed infection with another enteric pathogen than when they were the sole pathogens (Supplementary Table 8). Mixed infections with each DEC pathotype were significantly more common in cases than in controls for all pathotypes (EAEC, 96.0% vs 94.2%, *P* = .0018; tEPEC, 98.1% vs 97.5%, *P* = .0069; and aEPEC, 97.6% vs 94.6%, *P* = .0003). When trends were apparent, symptoms tended to be more marked when the DEC pathotype was part of a coinfection (Table 4). All deaths were reported in cases with coinfection. The frequency of coinfections was further evaluated to determine the dominant pair of a DEC pathotype and any of the most common enteric pathogens in cases and controls (Figure 3). In this analysis, coinfection with *Shigella*/EIEC was predominant and was consistently higher in cases (38%–48%) than in controls (28%–30%, *P* < .01). Notably, bloody diarrhea was not seen in any of the 6 episodes with STEC alone, 35 (31.3%) of the episodes with STEC plus *Shigella*/EIEC, and 26 (50%) of the episodes with *Shigella*/EIEC as the sole pathogen. Bloody diarrhea was observed more often when any of the other pathotypes was accompanied by *Shigella* vs those without concomitant *Shigella*, as follows: EAEC 289 (27.7%) vs 8 (7.1%), tEPEC 116 (27.1%) vs 1 (5%), and tEPEC 114 (27.4%) vs 2 (7.4%).

**Table 4.** Clinical Characteristics and Severity of Moderate-to-Severe Diarrhea Children With Either the Single Diarrheagenic *Escherichia coli* (DEC) Pathotype Detected or the Single DEC Pathotype Coinfected With Another Enteric Pathogen

Clinical Characteristic	EAEC Only^a^ n = 112	EAEC + Any^a^ n = 2639	tEPEC Only^a^ n = 20	tEPEC + Any^a^ n = 1023	aEPEC Only^a^ n = 27	aEPEC + Any^a^ n = 1070	STEC Only^a^ n = 6	STEC + Any^a^ n = 235
Duration of diarrheal episode, median (Q1–Q3), d	6 (4–8)	5 (4–8)	4 (3–6.25)	5 (4–8)	4 (2–6)	5 (3–8)	6.5 (4.5–8.5)	5 (3–8)
Maximum number of stools per day on worst day								
ȃ3	37 (33.0%)	630 (23.9%)	5 (25.0%)	272 (26.6%)	9 (33.3%)	250 (23.4%)	2 (33.3%)	56 (23.8%)
ȃ4–5	62 (55.4%)	1556 (59.0%)	10 (50.0%)	590 (57.7%)	14 (51.9%)	641 (59.9%)	4 (66.7%)	136 (57.9%)
ȃ6–10	13 (11.6%)	429 (16.3%)	4 (20.0%)	152 (14.9%)	3 (11.1%)	171 (16%)	0	37 (15.7%)
ȃ>10	0	24 (0.9%)	1 (5.0%)	9 (0.9%)	1 (3.7%)	8 (0.7%)	0	6 (2.6%)
Persistent diarrhea (>14 d)	13 (11.6%)	242 (9.2%)	1 (5.0%)	90 (8.8%)	1 (3.7%)	104 (9.7%)	0	26 (11.1%)
Blood in stool	8 (7.1%)	422 (16.0%)	1 (5.0%)	162 (15.8%)	2 (7.4%)	176 (16.4%)	0	47 (20%)
Maximum number of vomiting episodes on worst day								
ȃNone	65 (58.0%)	1415 (53.6%)	11 (55.0%)	560 (54.7%)	15 (55.6%)	594 (55.5%)	5 (83.3%)	131 (55.7%)
ȃ1	14 (12.5%)	229 (8.7%)	0	93 (9.1%)	1 (3.7%)	86 (8.0%)	0	23 (9.8%)
ȃ2–4	27 (24.1%)	847 (32.1%)	7 (35.0%)	313 (30.6%)	8 (29.6%)	339 (31.7%)	0	69 (29.4%)
ȃ≥5	6 (5.4%)	148 (5.6%)	2 (10.0%)	57 (5.6%)	3 (11.1%)	51 (4.8%)	1 (16.7%)	12 (5.1%)
Fever	24 (21.4%)	657 (24.9%)	3 (15.0%)	227 (22.2%)	3 (11.1%)	252 (23.6%)	2 (33.3%)	70 (29.8%)
Oral rehydration solution (given or prescribed)	112 (100.0%)	2569 (97.3%)	20 (100.0%)	987 (96.5%)	27 (100.0%)	1046 (97.8%)	6 (100%)	226 (96.2%)
Intravenous fluids (given or prescribed)	4 (3.6%)	186 (7.0%)	3 (15.0%)	70 (6.8%)	3 (11.1%)	72 (6.7%)	0	15 (6.4%)
Hospitalized	9 (8.0%)	165 (6.3%)	1 (5.0%)	62 (6.1%)	3 (11.1%)	54 (5.0%)	0	21 (8.9%)
Died	0	30 (1.1%)	0	12 (1.2%)	0	6 (0.6%)	0	2 (0.9%)

Abbreviations: aEPEC, atypical enteropathogenic *E. coli*; EAEC, enteroaggregative *E. coli*; STEC, Shiga toxin–producing *E. coli*; tEPEC, typical enteropathogenic *E. coli*.

“Only” denotes children with moderate-to-severe diarrhea (MSD) who had only this pathogen detected using TaqMan Array Card system quantitative polymerase chain reaction (TAC qPCR). “Any” denotes children with MSD who had the diarrheagenic *Escherichia coli* pathogen and any of the following detected using TAC qPCR: adenovirus 40/41, *Aeromonas* spp., astrovirus, toxigenic *Bacillus fragilis*, *Campylobacter* spp., *Cryptosporidium* spp., *Enterocytozoon bieneusi*, *Helicobacter pylori*, norovirus GI, norovirus GII, *Plesiomonas* spp., rotavirus, *Salmonella* spp., sapovirus, *Shigella* spp./Enteroinvasive *E. coli*, heat stable– or heat labile–producing enterotoxigenic *E. coli*.

## DISCUSSION

Testing the stool samples in VIDA using both conventional and TAC assays provided an opportunity to directly determine whether TAC enhanced the ability to detect pathogenic DEC strains among children with MSD participating in a large, controlled study in settings with high diarrheal disease burden. Our findings indicate that although the 3 DEC pathotypes evaluated were found far more commonly using TAC than the conventional assays, the fold-increase was similar among cases and controls. Neither method identified a significant association between a DEC pathotype and MSD. In fact, EAEC, aEPEC, and STEC were detected using TAC significantly more often in controls compared with cases, while the difference for tEPEC was insignificant. These findings also highlight the importance of using controls in studies of diarrhea etiology.

Numerous controlled studies have indicated that tEPEC is an important cause of community-acquired diarrhea in children from LMICs, particularly among infants aged ≤12 months living in Latin America [21–26]. Therefore, it was surprising that we did not detect an association between tEPEC and MSD at any site or in any age group. Particularly unexpected was the finding among infants at the Kenyan site, where a few years previously an association with MSD had been observed in GEMS using the same TAC qPCR assay and general geographic area [8, 12]. Nonetheless, other controlled studies have also failed to show a significant difference in the frequency of tEPEC in cases and controls, and some have suggested that the prevalence of tEPEC may be declining [10, 27–29], thus creating an incongruent picture of the role of tEPEC as a cause of diarrhea in LMICs.

Conflicting findings have also been reported concerning the association between MSD and the remaining DEC, aEPEC, EAEC, and STEC. The lack of a relationship that we observed in VIDA corroborates negative findings from recent studies that included Etiology, Risk Factors, and Interaction of Enteric Infections and Malnutrition and the Consequences for Child Health and Development project (MAL-ED) [10] and others [3, 10, 25, 30]. Among the 7 sites and 3 age strata in GEMS [23], EAEC was only associated with endemic MSD in children aged 12–23 months from Bangladesh [31–33]. In contrast, other studies have found an association with endemic diarrhea in children, especially prolonged episodes, for both aEPEC [23, 31–33] and EAEC [26, 34]. Although we have identified a reservoir among humans and ruminants, as seen in high-income countries [5], STEC was the least common pathotype, which is consistent with previous observations in LMICs [35, 36]. Moreover, STEC was not associated with MSD or hemorrhagic colitis, presentations seen in high-income settings, using *stx*_1_ and/or *stx*_2_ with or without inclusion of *eae* as a marker. The conflicting results for tEPEC, aEPEC, EAEC, and STEC raise questions about whether the gene targets currently used to identify these pathogens include some strains capable of causing diarrhea, but that additional factors must be present for full expression of disease.

Indeed, recent investigations have begun to uncover potential factors that may help to explain the drivers of DEC pathogenicity [37]. Genomic analyses of EPEC and EAEC have demonstrated considerable diversity in both core virulence loci and virulence plasmids, even within the same phylogenomic lineage, suggesting that these pathotypes have continued to acquire genetic changes since their initial acquisition of their defining features [38, 39]. New targets associated with disease severity that have not been included in current assays have been found [39, 40]. Expression of virulence factors is under the control of complex regulatory mechanisms derived from the host (eg, age, nutritional status [41], and genetic factors [37]), the organism, and environmental milieu such as intestinal microbiota, nutrients, and oxygen tension [39, 41, 42]. Correlations of these findings with isolate-specific clinical manifestations are being explored [43].

There is evidence to suggest that DEC induce intestinal inflammation that can lead to growth and nutritional faltering even in the absence of diarrheal disease [8, 25, 44–50], particularly during the first 2 years of life. In addition, both tEPEC and EAEC were associated with an increased risk of death in GEMS within 2–3 months after onset of the MSD episode [51]. Despite these observations, no DEC pathotype in VIDA was significantly associated with stunting among cases reexamined 2–3 months after enrollment. Cases with DEC were not more likely to die or to exhibit more pronounced illness when fever, blood in stool, duration of diarrhea, and vomiting were examined.

DEC pathotypes were identified much more often in mixed infections with other enteric pathogens than as the sole pathogen. Compared with sole infection, coinfection with other pathogens did not significantly enhance the symptomatology in MSD cases as has been reported elsewhere, with the exception that *Shigella* increased the occurrence of blood in stools for all pathotypes [13]. Of note, coinfection with *Shigella* was consistently high, especially among MSD cases.

A significant limitation in this study is that because TAC was performed directly on stool samples, DEC that meet criteria for multiple virulence targets may be identifying the genes on different microorganisms. Another limitation is that the high frequency of asymptomatic carriage and coinfections make it challenging to attribute clinical findings to DEC, and associations with MSD disease may be obscured.

In conclusion, we did not identify a role for EAEC, EPEC, or STEC in causing MSD at sites in sub-Saharan Africa. Given the diversity of the DEC strains, it is likely that particular strains or subtypes may cause disease. Future genomic analysis and investigations into the factors that regulate expression of virulence factors during diarrhea will be necessary to gain insight into the role of DEC in diarrheal disease in the African setting and elsewhere.

## Supplementary Data

Supplementary materials are available at *Clinical Infectious Diseases* online. Consisting of data provided by the authors to benefit the reader, the posted materials are not copyedited and are the sole responsibility of the authors, so questions or comments should be addressed to the corresponding author.

## Supplementary Material

ciad035_Supplementary_Data

## Notes

***Acknowledgments.*** The authors thank the families who participated in these studies, the clinical and field staff for their exceptional hard work and dedication, and the physicians, administration, and health officials at every site who generously provided facilities and support for the conduct of the study. The authors are grateful to Catherine Johnson, Chris Focht, and Nora Watson at the Emmes Company, LLC, for expert data management and reporting. Special thanks go to Carl Kirkwood, Duncan Steele, and Anita Zaidi at the Bill & Melinda Gates Foundation for helpful oversight, Kathy Neuzil for thoughtful suggestions, and the following members of our International Scientific Advisory Committee for providing insightful comments and guidance: Janet Wittes (chair), George Armah, John Clemens, Christopher Duggan, Stephane Helleringer, Ali Mokdad, James Nataro, and Halvor Sommerfelt.

***Disclaimer.*** The funding organization(s) had no role in the design, collection, analysis, or interpretation of data or in the writing of the manuscript. The findings and conclusions presented here are those of the authors and do not necessarily represent the official position of the Kenya Medical Research Institute, University of Maryland, US Centers for Disease Control and Prevention (CDC), nor any of the collaborating partners in this project.

***Financial support.*** This work was supported by the Bill & Melinda Gates Foundation (grant OPP1111236 and grant OPP1116751).

***Supplement sponsorship.*** This supplement was sponsored by the Bill & Melinda Gates Foundation. This study is based on research funded in part by grants from the Bill & Melinda Gates Foundation (OPP1111236/OPP1116751).

## References

[1] Robins-Browne RM , Holt KE, Ingle DJ, Hocking DM, Yang J, Tauschek M. Are *Escherichia coli* pathotypes still relevant in the era of whole-genome sequencing? Front Cell Infect Microbiol 2016; 6:141. 27917373 10.3389/fcimb.2016.00141 PMC5114240

[2] Byrne L , Adams N, Jenkins C. Association between Shiga toxin-producing *Escherichia coli* O157:H7 *stx* gene subtype and disease severity, England, 2009–2019. Emerg Infect Dis 2020; 26:2394–400. 32946720 10.3201/eid2610.200319 PMC7510717

[3] Kotloff KL , Nataro JP, Blackwelder WC, et al Burden and aetiology of diarrhoeal disease in infants and young children in developing countries (the Global Enteric Multicenter Study, GEMS): a prospective, case-control study. Lancet 2013; 382:209–22. 23680352 10.1016/S0140-6736(13)60844-2

[4] O’Ryan M , Prado V, Pickering LK. A millennium update on pediatric diarrheal illness in the developing world. Semin Pediatr Infect Dis 2005; 16:125–36. 15825143 10.1053/j.spid.2005.12.008

[5] Nataro JP , Kaper JB. Diarrheagenic *Escherichia coli*. Clin Microbiol Rev 1998; 11:142–201. 9457432 10.1128/cmr.11.1.142 PMC121379

[6] Barletta F , Ochoa TJ, Mercado E, et al Quantitative real-time polymerase chain reaction for enteropathogenic *Escherichia coli*: a tool for investigation of asymptomatic versus symptomatic infections. Clin Infect Dis 2011; 53:1223–9. 22028433 10.1093/cid/cir730 PMC3214587

[7] Bonkoungou IJ , Lienemann T, Martikainen O, et al Diarrhoeagenic *Escherichia coli* detected by 16-plex PCR in children with and without diarrhoea in Burkina Faso. Clin Microbiol Infect 2011; 18:901–6. 21985619 10.1111/j.1469-0691.2011.03675.x

[8] Fagerli K , Omore R, Kim S, et al Factors associated with typical enteropathogenic *Escherichia coli* infection among children <5 years old with moderate-to-severe diarrhoea in rural western Kenya, 2008–2012. Epidemiol Infect 2020; 148:e281. 33190663 10.1017/S0950268820002794 PMC7770376

[9] Iturriza-Gomara M , Jere KC, Hungerford D, et al Etiology of diarrhea among hospitalized children in Blantyre, Malawi, following rotavirus vaccine introduction: a case-control study. J Infect Dis 2019; 220:213–8. 30816414 10.1093/infdis/jiz084 PMC6581894

[10] Platts-Mills JA , Babji S, Bodhidatta L, et al Pathogen-specific burdens of community diarrhoea in developing countries: a multisite birth cohort study (MAL-ED). Lancet Glob Health 2015; 3:e564–75. 26202075 10.1016/S2214-109X(15)00151-5 PMC7328884

[11] Liu J , Platts-Mills JA, Juma J, et al Use of quantitative molecular diagnostic methods to identify causes of diarrhoea in children: a reanalysis of the GEMS case-control study. Lancet 2016; 388:1291–301. 27673470 10.1016/S0140-6736(16)31529-X PMC5471845

[12] Kotloff KL , Sow SO, Hossain MJ, et al Changing landscape of moderate-to-severe diarrhea among children in 3 sub-Saharan African countries following rotavirus vaccine introduction: the Vaccine Impact on Diarrhea in Africa (VIDA), in preparation.

[13] Mathew S , Smatti MK, Al Ansari K, Nasrallah GK, Al Thani AA, Yassine HM. Mixed viral-bacterial infections and their effects on gut microbiota and clinical illnesses in children. Sci Rep 2019; 9:865. 30696865 10.1038/s41598-018-37162-w PMC6351549

[14] Baqui AH , Yunus MD, Zaman K, Mitra AK, Hossain KM. Surveillance of patients attending a rural diarrhoea treatment centre in Bangladesh. Trop Geogr Med 1991; 43(1–2):17–22. 1750109

[15] Nasrin D , Liang Y, Powell H, et al Moderate-to-severe diarrhea and stunting among children younger than 5 years: findings from the Vaccine Impact on Diarrhea in Africa (VIDA) Study. Clin Infect Dis **2023**; 76(Suppl 1):S41–8. 10.1093/cid/ciac945 PMC10116556 37074430

[16] Kotloff KL , Blackwelder WC, Nasrin D, et al The Global Enteric Multicenter Study (GEMS) of diarrheal disease in infants and young children in developing countries: epidemiologic and clinical methods of the case/control study. Clin Infect Dis 2012; 55(Suppl 4):S232–45. 23169936 10.1093/cid/cis753 PMC3502307

[17] Panchalingam S , Antonio M, Hossain A, et al Diagnostic microbiologic methods in the GEMS-1 case/control study. Clin Infect Dis 2012; 55(Suppl 4): S294–302. 23169941 10.1093/cid/cis754 PMC3502308

[18] Kotloff KL , Nasrin D, Blackwelder WC, et al The incidence, aetiology, and adverse clinical consequences of less severe diarrhoeal episodes among infants and children residing in low-income and middle-income countries: a 12-month case-control study as a follow-on to the Global Enteric Multicenter Study (GEMS). Lancet Glob Health 2019; 7:e568–e84. 31000128 10.1016/S2214-109X(19)30076-2 PMC6484777

[19] Liu J , Gratz J, Amour C, et al Optimization of quantitative PCR methods for enteropathogen detection. PLoS One 2016; 11:e0158199. 10.1371/journal.pone.0158199 PMC4918952 27336160

[20] WHO Multicentre Growth Reference Study Group . WHO child growth standards based on length/height, weight and age. Acta Paediatr Suppl 2006; 450:76–85. 16817681 10.1111/j.1651-2227.2006.tb02378.x

[21] Levine MM , Ferreccio C, Prado V, et al Epidemiologic studies of *Escherichia coli* infections in a low socioeconomic level periurban community in Santiago, Chile. Am J Epidemiol 1993; 138:849–69. 8237973 10.1093/oxfordjournals.aje.a116788

[22] Scaletsky IC , Fabbricotti SH, Silva SO, Morais MB, Fagundes-Neto U. HEp-2-adherent *Escherichia coli* strains associated with acute infantile diarrhea, São Paulo, Brazil. Emerg Infect Dis 2002; 8:855–8. 12141974 10.3201/eid0808.010492 PMC2732515

[23] Alikhani MY , Mirsalehian A, Aslani MM. Detection of typical and atypical enteropathogenic *Escherichia coli* (EPEC) in Iranian children with and without diarrhoea. J Med Microbiol 2006; 55(Pt 9):1159–63. 16914644 10.1099/jmm.0.46539-0

[24] Behiry IK , Abada EA, Ahmed EA, Labeeb RS. Enteropathogenic *Escherichia coli* associated with diarrhea in children in Cairo, Egypt. ScientificWorldJournal 2011; 11:2613–9. 22262949 10.1100/2011/485381 PMC3254012

[25] Santos AKS , de Medeiros P, Bona MD, et al Virulence-related genes and coenteropathogens associated with clinical outcomes of enteropathogenic *Escherichia coli* infections in children from the Brazilian semiarid region: a case-control study of diarrhea. J Clin Microbiol 2019; 57:e01777–18. 10.1128/JCM.01777-18 PMC6440785 30728193

[26] Gonzales L , Joffre E, Rivera R, Sjöling Å, Svennerholm AM, Iñiguez V. Prevalence, seasonality and severity of disease caused by pathogenic *Escherichia coli* in children with diarrhoea in Bolivia. J Med Microbiol 2013; 62(Pt 11):1697–706. 23851188 10.1099/jmm.0.060798-0

[27] Estrada-Garcia T , Lopez-Saucedo C, Thompson-Bonilla R, et al Association of diarrheagenic *Escherichia coli* pathotypes with infection and diarrhea among Mexican children and association of atypical enteropathogenic *E. coli* with acute diarrhea. J Clin Microbiol 2009; 47:93–8. 19020055 10.1128/JCM.01166-08 PMC2620860

[28] Vilchez S , Reyes D, Paniagua M, Bucardo F, Möllby R, Weintraub A. Prevalence of diarrhoeagenic *Escherichia coli* in children from León, Nicaragua. J Med Microbiol 2009; 58(Pt 5):630–7. 19369525 10.1099/jmm.0.007369-0

[29] Franzolin MR , Alves RC, Keller R, et al Prevalence of diarrheagenic *Escherichia coli* in children with diarrhea in Salvador, Bahia, Brazil. Mem Inst Oswaldo Cruz 2005; 100:359–63. 16113883 10.1590/s0074-02762005000400004

[30] Ochoa TJ , Barletta F, Contreras C, Mercado E. New insights into the epidemiology of enteropathogenic *Escherichia coli* infection. Trans R Soc Trop Med Hyg 2008; 102:852–6. 18455741 10.1016/j.trstmh.2008.03.017 PMC2575077

[31] Nguyen RN , Taylor LS, Tauschek M, Robins-Browne RM. Atypical enteropathogenic *Escherichia coli* infection and prolonged diarrhea in children. Emerg Infect Dis 2006; 12:597–603. 16704807 10.3201/eid1204.051112 PMC3294699

[32] Afset JE , Bevanger L, Romundstad P, Bergh K. Association of atypical enteropathogenic *Escherichia coli* (EPEC) with prolonged diarrhoea. J Med Microbiol 2004; 53(Pt 11):1137–44. 15496393 10.1099/jmm.0.45719-0

[33] Lima AAM , Oliveira DB, Quetz JS, et al Etiology and severity of diarrheal diseases in infants at the semiarid region of Brazil: a case-control study. PLoS Negl Trop Dis 2019; 13:e0007154. 10.1371/journal.pntd.0007154 PMC6383952 30735493

[34] Sarantuya J , Nishi J, Wakimoto N, et al Typical enteroaggregative *Escherichia coli* is the most prevalent pathotype among *E. coli* strains causing diarrhea in Mongolian children. J Clin Microbiol 2004; 42:133–9. 14715743 10.1128/JCM.42.1.133-139.2004 PMC321701

[35] Breurec S , Vanel N, Bata P, et al Etiology and epidemiology of diarrhea in hospitalized children from low income country: a matched case-control study in Central African Republic. PLoS Negl Trop Dis 2016; 10:e0004283. 10.1371/journal.pntd.0004283 PMC4701495 26731629

[36] Lozer DM , Souza TB, Monfardini MV, et al Genotypic and phenotypic analysis of diarrheagenic *Escherichia coli* strains isolated from Brazilian children living in low socioeconomic level communities. BMC Infect Dis 2013; 13:418. 24010735 10.1186/1471-2334-13-418 PMC3846636

[37] Lima AAM , Medeiros P, Havt A. Enteroaggregative *Escherichia coli* subclinical and clinical infections. Curr Opin Infect Dis 2018; 31:433–9. 30063473 10.1097/QCO.0000000000000477

[38] Hazen TH , Kaper JB, Nataro JP, Rasko DA. Comparative genomics provides insight into the diversity of the attaching and effacing *Escherichia coli* virulence plasmids. Infect Immun 2015; 83:4103–17. 26238712 10.1128/IAI.00769-15 PMC4567640

[39] Boisen N , Østerlund MT, Joensen KG, et al Redefining enteroaggregative *Escherichia coli* (EAEC): genomic characterization of epidemiological EAEC strains. PLoS Negl Trop Dis 2020; 14:e0008613. 10.1371/journal.pntd.0008613 PMC7500659 32898134

[40] Izquierdo M , Lopez J, Gallardo P, Vidal RM, Ossa JC, Farfan MJ. Bacteria from gut microbiota associated with diarrheal infections in children promote virulence of Shiga toxin-producing and enteroaggregative *Escherichia coli* pathotypes. Front Cell Infect Microbiol 2022; 12:867205. 10.3389/fcimb.2022.867205 PMC9396624 36017363

[41] Modgil V , Chaudhary P, Bharti B, et al Prevalence, virulence gene profiling, and characterization of enteroaggregative *Escherichia coli* from children with acute diarrhea, asymptomatic nourished, and malnourished children younger than 5 years of age in India. J Pediatr 2021; 234:106–14.e5. 33713662 10.1016/j.jpeds.2021.03.010

[42] Gelalcha BD , Brown SM, Crocker HE, Agga GE, Kerro Dego O. Regulation mechanisms of virulence genes in enterohemorrhagic *Escherichia coli*. Foodborne Pathog Dis 2022; 19:598–612. 35921067 10.1089/fpd.2021.0103

[43] Hazen TH , Daugherty SC, Shetty AC, Nataro JP, Rasko DA. Transcriptional variation of diverse enteropathogenic *Escherichia coli* isolates under virulence-inducing conditions. mSystems 2017; 2:e00024–17. 10.1128/mSystems.00024-17 PMC5527300 28766584

[44] Nataro JP , Guerrant RL. Chronic consequences on human health induced by microbial pathogens: growth faltering among children in developing countries. Vaccine 2017; 35(49 Pt A):6807–12. 28549806 10.1016/j.vaccine.2017.05.035

[45] Rogawski ET , Liu J, Platts-Mills JA, et al Use of quantitative molecular diagnostic methods to investigate the effect of enteropathogen infections on linear growth in children in low-resource settings: longitudinal analysis of results from the MAL-ED cohort study. Lancet Glob Health 2018; 6:e1319–e28. 30287125 10.1016/S2214-109X(18)30351-6 PMC6227248

[46] Steiner TS , Lima AA, Nataro JP, Guerrant RL. Enteroaggregative *Escherichia coli* produce intestinal inflammation and growth impairment and cause interleukin-8 release from intestinal epithelial cells. J Infect Dis 1998; 177:88–96. 9419174 10.1086/513809

[47] Buskirk AD , Ndungo E, Shimanovich AA, et al Mucosal immune profiles associated with diarrheal disease severity in Shigella- and enteropathogenic *Escherichia coli*-infected children enrolled in the Global Enteric Multicenter Study. mBio 2022; 13:e0053822. 35924851 10.1128/mbio.00538-22 PMC9426439

[48] Opintan JA , Newman MJ, Ayeh-Kumi PF, et al Pediatric diarrhea in southern Ghana: etiology and association with intestinal inflammation and malnutrition. Am J Trop Med Hyg 2010; 83:936–43. 20889896 10.4269/ajtmh.2010.09-0792 PMC2946773

[49] Platts-Mills JA , Taniuchi M, Uddin MJ, et al Association between enteropathogens and malnutrition in children aged 6–23 mo in Bangladesh: a case-control study. Am J Clin Nutr 2017; 105:1132–8. 28381477 10.3945/ajcn.116.138800 PMC5402031

[50] Nasrin D , Blackwelder WC, Sommerfelt H, et al Pathogens associated with linear growth faltering in children with diarrhea and impact of antibiotic treatment: the Global Enteric Multicenter Study. J Infect Dis 2021; 224(12 Suppl 2):S848–55. 34528677 10.1093/infdis/jiab434 PMC8958895

[51] Levine MM , Nasrin D, Acacio S, et al Diarrhoeal disease and subsequent risk of death in infants and children residing in low-income and middle-income countries: analysis of the GEMS case-control study and 12-month GEMS-1A follow-on study. Lancet Glob Health 2020; 8:e204–e14. 31864916 10.1016/S2214-109X(19)30541-8 PMC7025325

