# Supplementary material for: Epidemiology of Enteroaggregative, Enteropathogenic, and Shiga Toxin–Producing Escherichia coli Among Children Aged <5 Years in 3 Countries in Africa, 2015–2018: Vaccine Impact on Diarrhea in Africa (VIDA) Study
Source: Clin Infect Dis. 2023 Apr 19;76(Suppl 1):S77–86. doi: 10.1093/cid/ciad035 (PMC10116530; doi:10.1093/cid/ciad035)
Supplement: ciad035_Supplementary_Data [file ciad035_supplementary_data.zip › Ochieng_Ecoli_Supplemental_revised-clean 6Jan2022.docx]

**Appendix**

Epidemiology of Enteroaggregative, Enteropathogenic and Shiga toxin-producing *Escherichia coli* among Children <5 Years of Age in Three Countries in Africa, 2015-2018: Vaccine Impact on Diarrhea in Africa (VIDA) Study

John B. Ochieng, Helen Powell, Ciara E. Sugerman, Richard Omore, Billy Ogwel, Jane Juma, Alex O. Awuor , Samba O. Sow, Doh Sanogo, Uma Onwuchekwa, Adama Mamby Keita, Awa Traoré, Henry Badji, M. Jahangir Hossain, Joquina Chiquita M. Jones, Irene N. Kasumba, Dilruba Nasrin, Anna Roose, Yuanyuan Liang, Leslie P. Jamka, Martin Antonio, James A. Platts-Mills, Jie Liu, Eric R. Houpt, Eric D. Mintz, Elizabeth Hunsperger, Clayton O. Onyango, Nancy Strockbine, Marc-Alain Widdowson, Jennifer R. Verani, Sharon M. Tennant, Karen L. Kotloff

1. **Supplementary Tables**

**Supplementary Table 1**: Data cleaning and analytic methods used for the propensity score analysis of stunting

**Supplementary Table 2**: Comparing TAC and conventional PCR method in detection of EAEC, EPEC and STEC from MSD cases and control

**Supplementary Table 3**: Taqman Array Card (TAC) cycle threshold (Ct) values for selected diarrheagenic *E. coli* pathotypes among children under 5 years old with moderate-to-severe diarrhea (MSD) and their MSD-free controls, for all positive results (Ct value <35).

**Supplementary Table 4**: Proportion of cases and controls meeting the definition of STEC according to the Shiga toxin genotype status (*stx*1 and/or *stx*2) and the presence or absence of *eae* detected by TAC qPCR in VIDA

**Supplementary Table 5**: Crude association of TAC detected EPEC, EAEC and STEC with stunting, at enrollment and at ~60 days follow-up, among MSD cases

**Supplementary Table 6**: Association of EPEC, EAEC and STEC with stunting, at ~60 days follow-up, among MSD children <5 years old following propensity score matching

**Supplementary Table 7**: Results for each of the propensity score matching methods used in the STEC analyses

**Supplementary Table 8**: Test of two independent proportions using Yates continuity correction to compare co-infection in cases and controls

1. **Supplementary Figures**

**Supplementary Figure 1**: CRF09: Memory Aid Score Sheet

**Supplementary Figure 2**: Taqman Array Card (TAC) cycle threshold (Ct) distribution for enteroaggregative *Escherichia coli* (EAEC), typical enteropathogenic *E. coli* (tEPEC), atypical enteropathogenic *E. coli (*aEPEC), and Shiga toxin -producing *E. coli* (STEC) among children under 5 years old with moderate-to-severe diarrhea (MSD) and their MSD-free controls, for all positive results (Ct value <35).

**Supplementary Figure 3**: CRF04A: Enrolment of Cases - H (ECH)

**Supplementary Figure 4**: CRF04B: Enrollment of Controls - M (ECM)

| Supplementary Table 1: Data cleaning and the analytic methods used for the propensity score analysis of stunting | |
| --- | --- |
| Method | **Description** |
| Data cleaning for the stunting analyses only* | - Cases who had missing or implausible height/length measurements were excluded from the analysis. - The following criteria was used to identify implausible values: - \|HAZ\| > 6 and change in HAZ > 3; - A height/length gain of >8 cm from enrolment to follow-up 50-60 days later or a gain of >10 cm from enrolment to follow-up 61-90 days later among those ≤ 6 months; - A height/length gain of >4 cm from enrolment to follow-up 50-60 days later or a gain of >6 cm from enrolment to follow-up 61-90 days later among those >6 months; - A height/length which decreased by more than 1.5 cm from enrolment to follow-up. |
| Propensity score analysis of stunting | - The propensity score was calculated separately for each pathotype. - The following variables were included in the PS matching: stunting at enrolment, time between enrolment and follow-up visit, age, gender, site, caretaker education, breastfeeding in the weeks prior to infection, cooking fuels, assets, access to improved water, access to improved sanitation, finished floors, more than 3 people per sleeping room, more than 2 children under the age of 5, and indicators of other enteric infections or pneumonia as detailed in the CRFs (**Supplementary Figures 3 and 4**). - We initially used one-to-one nearest neighbor matching without replacement and examined whether or not a good match was achieved by confirming that the percentage bias between the standardized difference between the pathotype positive and negative MSD cases was less than an absolute value of 10 for each matching variable. - If a good match could not be achieved across all variables, we examined the results using one-to-one nearest neighbor matching without replacement with a caliper of 0.05, and finally one-to-one nearest neighbor matching with replacement, to ensure that the results were robust to those variables where a good match could not be achieved. |
| * The WHO child growth standards are only applicable to children under the age of 5 years. Therefore, any child whose age exceeded 59 months at follow-up had their follow-up HAZ-score estimated using a pseudo age of 59 months. | |

| Supplementary Table 2: Comparing TAC and conventional PCR method in detection of EAEC, EPEC and STEC from MSD cases and control | | | | |
| --- | --- | --- | --- | --- |
| Pathotypes | **TAC results** | **Conventional Results** | | |
|  |  | **Positive (%)** | **Negative (%)** | **Total** |
| EAEC – Cases  (n = 4719) | Positive | 672 (23.9) | 2,145 (76.1) | 2,817 |
|  | Negative | 83 (4.4) | 1,819 (95.6) | 1,902 |
|  |  |  |  |  |
| EAEC – Controls  (n = 4733) | Positive | 804 (26.0) | 2,284 (74.0) | 3,088 |
|  | Negative | 62 (3.8) | 1,583 (96.2) | 1,645 |
|  |  |  |  |  |
| tEPEC – Cases  (n = 4790) | Positive | 286 (26.6) | 789 (73.4) | 1,075 |
|  | Negative | 210 (5.7) | 3,505 (94.3) | 3,715 |
|  |  |  |  |  |
| tEPEC – Controls  (n = 4795) | Positive | 247 (22.7) | 839 (77.3) | 1,086 |
|  | Negative | 307 (8.3) | 3,402 (91.7) | 3,709 |
|  |  |  |  |  |
| aEPEC – Cases  (n = 4,788) | Positive | 53 (4.7) | 1,074 (95.3) | 1,127 |
|  | Negative | 48 (1.3) | 3,613 (98.7) | 3,661 |
|  |  |  |  |  |
| a EPEC – Controls  (n = 4,789) | Positive | 55 (4.2) | 1,263 (95.8) | 1,318 |
|  | Negative | 84 (2.4) | 3,387 (97.6) | 3,471 |
|  |  |  |  |  |
| STEC – Cases  (n = 4,792) | Positive | 1 (0.4) | 246 (99.6) | 247 |
|  | Negative | 3 (0.1) | 4,542 (99.9) | 4,545 |
|  |  |  |  |  |
| STEC – Controls (n = 4,786) | Positive | 2 (0.4) | 449 (99.6) | 451 |
|  | Negative | 3 (0.1) | 4,332 (99.9) | 4,335 |
| Abbreviations: MSD, moderate-to-severe diarrhea; EAEC, enteroaggregative *E. coli*; tEPEC, typical enteropathogenic *E. coli*; aEPEC, atypical enteropathogenic *E. coli*; STEC, Shiga toxin-producing *E. coli*; TAC, Taqman Array Card; qPCR, quantitative polymerase chain reaction, Ct, cycle threshold | | | | |

**Supplementary Table 3**: **Taqman Array Card (TAC) cycle threshold (Ct) values for selected diarrheagenic *E. coli* pathotypes among children under 5 years old with moderate-to-severe diarrhea (MSD) and their MSD-free controls, for all positive results (Ct value <35).**

| **Pathogen** |  |  | **Case** |  |  | **Control** |  |  |  |
| --- | --- | --- | --- | --- | --- | --- | --- | --- | --- |
|  |  |  | **Median Ct value [IQR]** |  |  | **Median Ct Value [IQR]** |  |  | **P-value** |
| EAEC |  |  | 26.57 [21.52-31.00] |  |  | 26.52 [21-30.74] |  |  | 0.7117 |
| aEPEC |  |  | 29.42 [25.56-32.71] |  |  | 29.28 [25.43-32.35] |  |  | 0.4242 |
| tEPEC |  |  | 26.34 [20.88-31.29] |  |  | 27.25 [22.99-31.53] |  |  | **0.0001** |
| STEC |  |  | 31.78 [29.34-33.35] |  |  | 31.98 [29.94-33.09] |  |  | 0.9932 |

Abbreviations: IQR, interquartile range; EAEC, enteroaggregative *Escherichia coli*; tEPEC. typical enteropathogenic *E. coli*, aEPEC, atypical enteropathogenic *E. coli,* STEC, Shiga toxin -producing *E. coli.*

| **Supplementary Table 4. Proportion of cases and controls meeting the definition of STEC according to the Shiga toxin genotype status (*stx*1 and/or *stx*2) and the presence or absence of *eae* detected by TAC qPCR in VIDA** | | | | | |
| --- | --- | --- | --- | --- | --- |
|  | Cases  (n = 247) | |  | Controls  (n = 451) | |
|  | No. | (%) |  | No. | (%) |
| *stx*1 alone | 93 | (37.7) |  | 147 | (32.6) |
| *eae* negative | 35 | (14.2) |  | 42 | (9.3) |
| *eae* positive | 58 | (23.5) |  | 105 | (23.3) |
| *stx*2 alone | 91 | (36.8) |  | 159 | (34.3) |
| *eae* negative | 37 | (15.0) |  | 40 | (8.9) |
| *eae* positive | 54 | (21.9) |  | 119 | (26.4) |
| *stx*1 and *stx*2 | 63 | (25.5) |  | 145 | (32.2) |
| *eae* negative | 14 | (5.7) |  | 31 | (6.9) |
| *eae* positive | 48 | (19.4) |  | 114 | (25.3) |
| Abbreviations: VIDA, Vaccine Impact on Diarrhea in Africa; STEC, Shiga toxin-producing *E. coli;* TAC, Taqman Array Card; qPCR, quantitative polymerase chain reaction | | | | | |

| Supplementary Table 5: Crude association of TAC detected EPEC, EAEC and STEC with stunting, at enrollment and at ~60 days follow-up, among MSD cases | | | | | | | | | | | | | |
| --- | --- | --- | --- | --- | --- | --- | --- | --- | --- | --- | --- | --- | --- |
| Parameter | MSD cases (N=4,603)^#^ | | | | | | | | | | | | |
|  | EAEC | |  | tEPEC | |  | aEPEC | |  | STEC | |  |  |
|  | Positive  n=2,681 | Negative  n=1,812 | *p-*value^a^ | Positive  n=1,013 | Negative  n=3,553 | *p*-value ^a^ | Positive  n=1,086 | Negative  n=3,478 | *p*-value ^a^ | Positive  n=235 | Negative  n=4,330 | *p-*value ^a^ |  |
| Stunted at enrollment, n (%) | 601 (22.4%) | 376 (20.8%) | 0.197 | 238 (23.5%) | 760 (21.4%) | 0.166 | 250 (23.0%) | 747 (21.5%) | 0.302 | 70 (29.8%) | 927 (21.4%) | **0.003** |  |
| Stunted at follow-up, n (%) | 737 (27.5%) | 447 (24.7%) | **0.038** | 291 (28.7%) | 919 (25.9%) | 0.075 | 300 (27.6%) | 909 (26.1%) | 0.352 | 77 (32.8%) | 1,132 (26.1%) | **0.031** |  |
| Abbreviations: MSD, moderate-to-severe diarrhea; EAEC, enteroaggregative *E. coli*; tEPEC, typical enteropathogenic *E. coli*; aEPEC, atypical enteropathogenic *E. coli*; STEC, Shiga toxin-producing *E. coli*  ^a^ Chi-square tests were used to estimate P-values; significant values are shown in bold.  ^#^110 had missing EAEC; 37 had missing tEPEC; 39 had missing aEPEC; and 38 had missing STEC. | | | | | | | | | | | | | |

| Supplementary Table 6: Association of EPEC, EAEC and STEC with stunting, at ~60 days follow-up, among MSD children <5 years old following propensity score matching | | | | | |
| --- | --- | --- | --- | --- | --- |
| DEC pathotypes | Unmatched | | Matched | | |
|  | qPCR | Stunting at follow-up before matching | Risk of stunting at follow-up | Difference in stunting at follow-up [95% CI] | p-value^a^ |
| EAEC  n=4,274 | Positive (n=2,550)^b^ | 27.4% | 24.2% | -0.12%  [-2.22%,1.98%] | 0.914 |
|  | Negative (n=1,724) | 24.3% | 24.3% |  |  |
| tEPEC  n=4,274 | Positive (n=937) | 28.6% | 28.6% | 0.10%  [-2.95%, 3.16%] | 0.945 |
|  | Negative (n=3,337) | 25.5% | 28.5% |  |  |
| aEPEC  n=4,274 | Positive (n=1,006) | 27.3% | 27.3% | -0.10%  [-3.82%, 3.62%] | 0.958 |
|  | Negative (n=3,269) | 25.8% | 27.4% |  |  |
| STEC  n=4,274 | Positive (n=216) | 31.9% | 31.9% | -1.85%  [-8.14%, 4.43%] | 0.564 |
|  | Negative (n=4,058) | 25.9% | 33.8% |  |  |
| Abbreviations: MSD, moderate-to-severe diarrhea; EAEC, enteroaggregative *E. coli*; tEPEC, typical enteropathogenic *E. coli*; aEPEC, atypical enteropathogenic *E. coli*; STEC, Shiga toxin-producing *E. coli*; TAC, Taqman Array Card; qPCR, quantitative polymerase chain reaction, Ct, cycle threshold  ^a^Bootstrap-based  ^b^Since the number of positive EAEC is larger than the number of negative EAEC, 1,724 positive EAEC were used after the propensity score matching. | | | | | |

| Supplementary Table 7: Results for each of the propensity score matching methods used in the STEC analyses | | | | | | | | | | |
| --- | --- | --- | --- | --- | --- | --- | --- | --- | --- | --- |
|  | 1-to-1 matching without replacement | |  | 1-to-1 matching without replacement and a caliper | |  | 1-to-1 matching without replacement | | |  |
| STEC TAC PCR | Risk of stunting at follow-up | Difference in stunting at follow-up [95% CI] | p-  value | Risk of stunting at follow-up | Difference in stunting at follow-up  [95% CI] | p-  value | Risk of stunting at follow-up | Difference in  stunting at follow-up  [95% CI] | p-  value | |
| Positive | 31.9% | -1.9% [-8.1, 4.4] | 0.564 | 31.6% | -1.7% [-8.5, 4.8] | 0.581 | 31.9% | 0 [-10.3, 10.3] | 1.000 | |
| Negative | 33.8% |  |  | 33.5% |  |  | 31.9% |  |  |  |
| Abbreviations: STEC, Shiga toxin-producing *E. coli*; TAC, Taqman Array Card; qPCR, quantitative polymerase chain reaction, Ct, cycle threshold | | | | | | | | |  | |

| Supplementary Table 8: Test of two independent proportions using Yates continuity correction to compare co-infection in cases and controls | | | | | | | | |
| --- | --- | --- | --- | --- | --- | --- | --- | --- |
|  |  | Multiple pathogens in cases | |  | Multiple pathogens in controls | |  | p-value |
| Pathotype |  | Number | % |  | Number | % |  |  |
| EAEC |  | 2706/2818 | 96.0 |  | 2912/3090 | 94.2 |  | 0.0018 |
|  |  |  |  |  |  |  |  |  |
| tEPEC |  | 1057/1077 | 98.1 |  | 1059/1086 | 97.5 |  | 0.0069 |
|  |  |  |  |  |  |  |  |  |
| aEPEC |  | 1100/1127 | 97.6 |  | 1250/1321 | 94.6 |  | 0.0003 |
|  |  |  |  |  |  |  |  |  |
| STEC |  | 241/247 | 97.6 |  | 435/451 | 96.5 |  | 0.5604 |
| Abbreviations: EAEC, enteroaggregative *E*. *coli*; tEPEC, typical enteropathogenic *E*. *coli*; aEPEC, atypical enteropathogenic *E*. *coli*; STEC, Shiga toxin-producing *E*. *coli* | | | | | | | | |
