# Supplementary figures and images for: Epidemiology of Enteroaggregative, Enteropathogenic, and Shiga Toxin–Producing Escherichia coli Among Children Aged <5 Years in 3 Countries in Africa, 2015–2018: Vaccine Impact on Diarrhea in Africa (VIDA) Study
Source: Clin Infect Dis. 2023 Apr 19;76(Suppl 1):S77–86. doi: 10.1093/cid/ciad035 (PMC10116530; doi:10.1093/cid/ciad035)

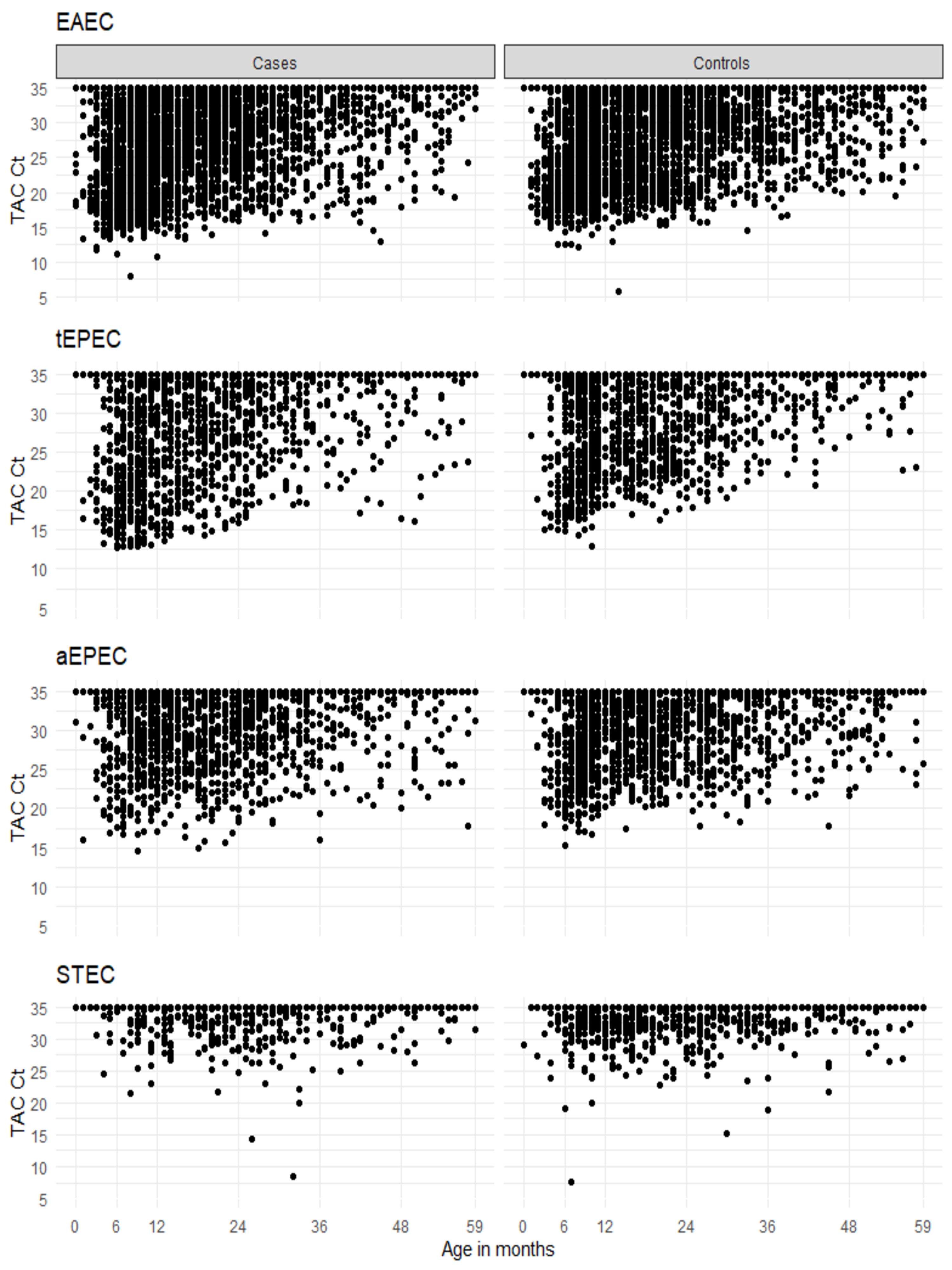

Supplement: ciad035_Supplementary_Data [file ciad035_supplementary_data.zip › Ochieng_Ecoli_supplemental figure 2.tiff]
